# Supplementary material for: A Global Screen for Assembly State Changes of the Mitotic Proteome by SEC-SWATH-MS
Source: Cell Syst. 2020 Feb 26;10(2):133–155.e6. doi: 10.1016/j.cels.2020.01.001 (PMC7042714; doi:10.1016/j.cels.2020.01.001)

Q7Z4S6 | KI21A_HUMAN | KIF21A KIAA1708 KIF2
Monomer MW [kDa]: 187.179 Monomer expected elution fraction: 35

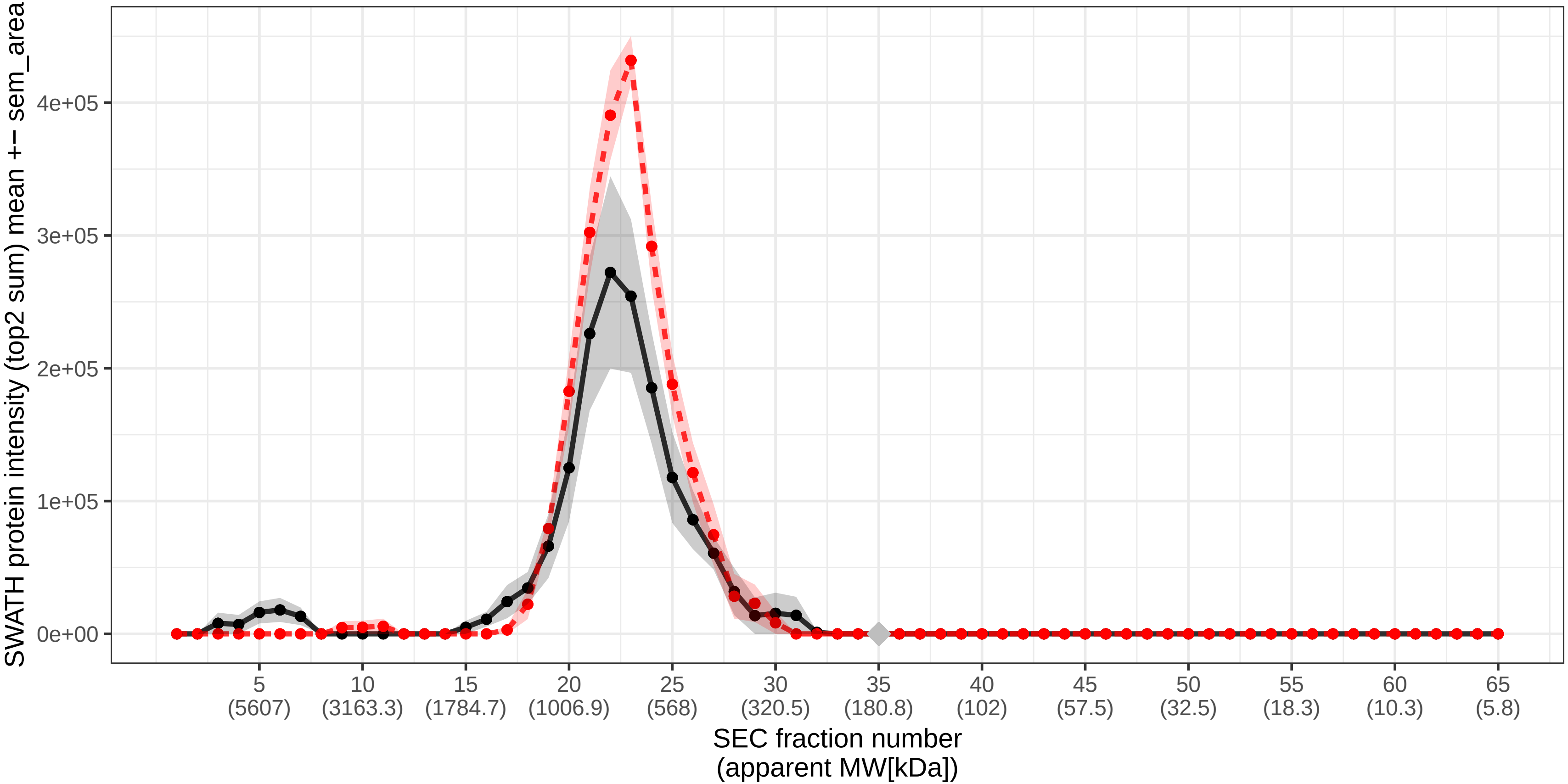

Supplement: Data S1. SEC-SWATH-MS Protein Chromatograms, Related to Figure 1 [file mmc6.zip › SECchrom_Q7Z4S6_KI21A_HUMAN_KIF21A_KIAA1708_KIF2.pdf]
